# Cytokines in inflammatory bowel disease

**DOI:** 10.1080/09629359791785

**Published:** 1997-04

**Authors:** P. L. Beck, J. L. Wallace

**Affiliations:** Intestinal Disease Research Unit Departments of Medicine and Pharmacology University of Calgary Alberta Calgary Canada

## Abstract

Over the past decade, much has been learned regarding the role of various cytokines in the pathogenesis of inflammatory bowel disease. Several cytokine ‘knockout’ models in mice have been shown to develop colitis, while alterations in the production of various cytokines has been documented in human Crohn's disease and ulcerative colitis. In recent years, attempts have been made to treat these diseases through modulation of cytokine production or action. This review focuses on the cytokines that have been implicated in the pathogenesis of inflammatory bowel disease. The evidence for and against a role for particular cytokines in intestinal inflammation is reviewed, as is the experimental and clinical data suggesting that cytokines are rational targets for the development of new therapies.


Mediators of Inflammation, **6**, 95–103 (1997)

### Cytokines in inflammatory bowel disease

P. L. Beck and J. L. Wallace[CA]

Intestinal Disease Research Unit, Departments of Medicine and Pharmacology, University of Calgary, Calgary, Alberta, Canada

[CA]Corresponding Author
Tel: (+1) 403 220 4539
Fax: (+1) 403 270 3353
Email: wallacej@acs.ucalgary.ca

**OVER the past decade, much has been learned regarding the role of various cytokines in the pathogenesis of inflammatory bowel disease. Several cytokine 'knockout' models in mice have been shown to develop colitis, while alterations in the production of various cytokines has been documented in human Crohn's disease and ulcerative colitis. In recent years, attempts have been made to treat these diseases through modulation of cytokine production or action. This review focuses on the cytokines that have been implicated in the pathogenesis of inflammatory bowel disease. The evidence for and against a role for particular cytokines in intestinal inflammation is reviewed, as is the experimental and clinical data suggesting that cytokines are rational targets for the development of new therapies.**

**Key words:** Crohn's disease, Interferons, Interleukins, Intestine, Tumour necrosis factor, Ulcerative colitis

### Introduction

'Inflammatory bowel disease' (IBD) is an umbrella term used to describe at least two distinct diseases: ulcerative colitis (UC) and Crohn's disease (CD). UC and CD are chronic inflammatory conditions affecting the gastrointestinal tract and are typified by unpredictable periods of remission and relapse. UC affects the large intestine and is usually limited to the mucosal layer. CD is a transmural disease (extending to the depth of the muscularis externae) and can occur anywhere in the gastrointestinal tract, although it most frequently affects the terminal ileum and large intestine.

Current treatment of IBD, which is modestly effective at best, primarily involves the use of corticosteroids, 5-amino salicylic acid, and to a lesser extent, immunosuppressants and antimicrobials. The pathogenesis of IBD remains poorly understood, hampering efforts to develop more effective treatments. It is generally believed that an impaired mucosal immune response to luminal microbes leads to an unrelenting inflammatory response with the generation of non-specific 'bystander' injury to the intestinal tissue. Considerable research in recent years has been directed at further understanding the mechanisms involved in regulating the inflammatory response in IBD. Several mediators are thought to play a role in this response and/or to contribute to the tissue injury and generation of symptoms, including nitric oxide, histamine, eicosanoids and cytokines. Given the key role of several cytokines in regulating mucosal immune responses, this family of mediators has received special attention by IBD researchers in the past decade. This review focuses on the emerging evidence for a role for various cytokines in the pathogenesis of IBD, and the possibility that these mediators represent rationale targets for therapeutic intervention. These cytokines can be divided into two categories: those that upregulate and those that downregulate the inflammatory response.

### Pro-inflammatory Cytokines

### Interleukin-1 (IL-1)

IL-1 is released early in the inflammatory cascade and has numerous pro-inflammatory actions (Table 1). IL-1 is produced by a wide array of cells, including macrophages, monocytes, endothelial cells and fibroblasts.[1] Two receptors for IL-1 have been identified. Type I receptors are found primarily on T lymphocytes and fibroblasts, while type II receptors have been identified on B lymphocytes, macrophages and monocytes.[1] IL-1 receptor antagonist (IL-1ra) is a naturally occurring antagonist to both IL-1 receptors that is produced by many of the same

© 1997 Rapid Science Publishers

**Table 1**. Actions of pro-inflammatory cytokines and relevance to IBD

| Cytokine | Actions on cells and mediators | Relevance to IBD |
|---|---|---|
| IL-1 | Promotes<br>• T cell activation<br>• NK cell activation<br>• B cell proliferation<br>Upregulates<br>• adhesion molecule expression<br>• eicosanoid and nitric oxide production | Increased levels in UC, CD and pouchitis and in animal models of colitis<br>Experimental colitis can be improved by treatment with IL-1 receptor antagonist and exacerbated by treatment with an antibody to IL-1 receptor antagonist |
| IL-2 | Promotes<br>• Th1 lymphocyte activation<br>• macrophage activation<br>• intraepithelial lymphocyte proliferation | IL-2 knockout mice develop colitis<br>Abnormal IL-2-induced T cell proliferation in UC<br>Decreased IL-2 levels in UC<br>Increased IL-2 mRNA in CD, not UC |
| IL-5 | Promotes<br>• eosinophil recruitment and differentiation | Increased IL-5 mRNA in CD, increased IL-5 production in UC |
| IL-6 | Promotes<br>• activation of T cells<br>• differentiation of B cells<br>• induction of hepatic acute phase reactants | Increased IL-6 levels in UC, CD and pouchitis<br>Levels of IL-6 correlate with disease activity and may predict relapses of CD |
| IL-8 | Promotes<br>• neutrophil recruitment and activation | Increased IL-8 levels in UC, CD and pouchitis |
| IL-12 | Promotes<br>• natural killer cell and cytotoxic T lymphocyte activity | Increased IL-12 levels in IBD (CD > UC) |
| TNFα | Promotes<br>• leukocyte adherence (through elevated adhesion molecule expression)<br>• activation of neutrophils, eosinophils, macrophages, lymphocytes | Increased TNFα levels in UC and CD<br>Anti-TNFα antibodies reduce inflammation in CD |
| IFNα | Promotes<br>• MHC expression<br>• NK and antibody-dependent cytotoxicity<br>• macrophage activation<br>Inhibits<br>• cell growth and differentiation | Increased IFNα mRNA in lamina propria cells from CD patients<br>IFNα therapy for CD appears to be of little benefit |

Abbreviations: NK, natural killer; MHC, major histocompatability complex; UC, ulcerative colitis; CD, Crohn's disease; IBD, inflammatory bowel disease.

cells that produce IL-1. IL-1 activity is, therefore, determined by the balance between its levels and those of IL-1ra at any target cell or tissue.[1]

Elevated levels of IL-1 activity in plasma and tissue have been demonstrated in both CD and UC, as well as in several experimental models of colitis.[2–9] Furthermore, the ratio of IL-1:IL-1ra is increased in CD and UC but not in self-limited colitis[8] (Fig. 1). The pivotal role of IL-1 and IL-1ra in regulating colonic inflammation has been demonstrated by the observation that administration of recombinant IL-1ra attenuates the inflammatory process in three different animal models of colitis.[10–12] The importance of IL-1ra was further demonstrated in a rabbit model of immune-complex colitis, where inflammation of the colon was exacerbated by the administration of neutralizing antibodies directed against IL-1ra[13] (Fig. 2).

Sher *et al.* found that in patients with CD, IL-1 and IL-8 levels were elevated in inflamed and normal colonic tissue.[14] Thus, cytokine activation may occur prior to the development of macroscopically visible damage or may simply involve the entire region of the gut when only limited macroscopic disease exists. A similar pattern was also noted in pouchitis (inflammation of a surgically constructed ileal reservoir). Levels of IL-1, IL-6 and IL-8 were increased in pouchitis, but levels of IL-1 and IL-8 were also increased in non-inflamed pouches relative to normal ileal mucosa.[15]

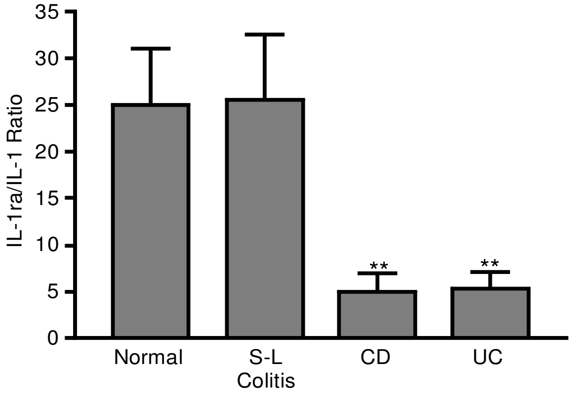

FIG. 1. Ratio of IL-1 receptor antagonist (IL-1ra) to IL-1 levels in mucosal biopsies from normal individuals, patients with self-limiting (S-L) colitis, Crohn's disease (CD) and ulcerative colitis (UC). The ratio is significantly (**$P < 0.01$) depressed in patients with UC and CD relative to the other two groups. Prepared using the data Casini-Raggi et al.[8] with permission of the authors.

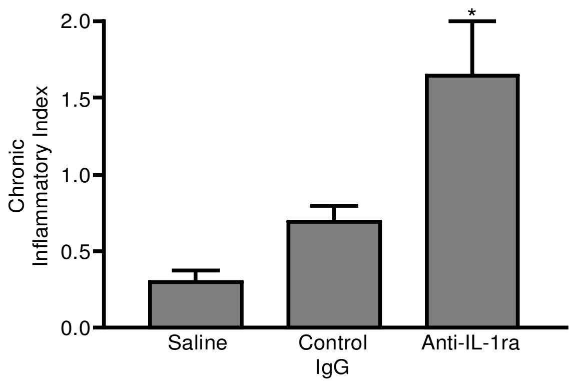

FIG. 2. Effects of treatment with an antibody directed against IL-1 receptor antagonist (IL-1ra) on severity of chronic inflammation in a rabbit model of colitis. Treatment with anti-IL-1ra significantly increased the severity of colitis relative to rabbits treated with a control antibody or to healthy rabbits receiving only saline (* $P < 0.03$). Prepared using the data of Cominelli et al.[13] with permission of the authors.

Therapy directed at regulating IL-1 activity appears promising. Cominelli and co-workers[16] reported that an inhibitor of IL-1 and TNFα synthesis (CGP 47969A) significantly decreased the severity of inflammation in a rabbit model of colitis. Clinical trials of recombinant IL-1ra were initiated a number of years ago, but were not completed due to disappointing results from trials of this cytokine unrelated to IBD. For several years, nicotine has been suggested to decrease activity in UC and recently it has been found to be effective in the treatment of active UC (but not in the maintenance of remission).[17,18] The mechanism of action of nicotine in the treatment of UC is not well understood, but it has been suggested to be due to its ability to suppress the production of IL-1 and TNFα.[19]

### Interleukin-2 (IL-2)

IL-2 is a key factor in the regulation of T lymphocyte proliferation.[1] It also influences proliferation of B lymphocytes and natural killer (NK) cells. High and low affinity receptors for IL-2 have been identified on several cells.[1–3] IL-2 levels and IL-2 responses are increased in CD, but decreased in UC.[20,21] In peripheral blood monocytes isolated from paediatric patients with IBD, indomethacin increased IL-2 production in monocytes from CD patients but not in those from UC patients.[22] A potential role of IL-2 in the pathogenesis of IBD was suggested when two patients with CD received high-dose IL-2 for the treatment of renal cell carcinoma[23] and both experienced a flare in CD symptoms requiring immediate bowel resection.[23]

In terms of IL-2-related therapy, cyclosporin A, FK506, and rapamycin are all inhibitors of lymphocyte activation and decrease IL-2 synthesis and/or IL-2 responsiveness. Cyclosporin A has been shown to exhibit some efficacy in the treatment of IBD,[24,25] while FK506 has shown promise in an animal model of colitis.[26]

### Interleukin-5 (IL-5)

IL-5 is among the most potent chemotaxins for eosinophils yet described, and can enhance immunoglobulin A secretion by B lymphocytes.[1–3] In a study looking at early recurrence of CD following resection, Dubucquoi *et al.* found that eosinophil infiltration was more pronounced in areas with endoscopically visible disease compared with normal-appearing tissue, and that increased numbers of eosinophils were associated with elevated IL-5 mRNA expression.[27] However, more direct evidence for a role of IL-5 in IBD is lacking.

Only one study has examined the role of IL-5 in experimental colitis, yielding results that were inconclusive. The severity of dextran sulphate sodium-induced colitis in mice in which the gene for IL-5 had been deleted (IL-5 knock-out mice) was found not to differ from that in normal mice.[28] Given the high likelihood that there is redundancy in terms of the number of mediators that can produce the pro-inflammatory effects ascribed to IL-5, and that there may be up-regulation of the production of these other mediators as a means of compensating for the lack of IL-5 in the knock-out mice, one cannot draw any firm conclusions from this study regarding the role of IL-5 in colitis.

### Interleukin-6 (IL-6)

IL-6 appears to be one of the most important cytokines in the inflammatory response. The main actions of IL-6 include activation of T and B lymphocytes and induction of hepatic acute phase reactants.[29] Previous studies noted elevations of IL-6 in both CD and UC and more recently in pouchitis.[3,14,15,30] Serum levels of IL-6 and soluble IL-6 receptor as well as tissue levels of IL-6 appear to correlate well with disease activity in both CD and UC.[31,32] Recently, a group from Belgium found that serum IL-6 levels in patients with inactive CD, followed for one year, correctly predicted whether a patient would relapse in 29 of 32 cases.[33]

### Interleukin-8 (IL-8)

IL-8 is a potent chemotaxin for neutrophils.[1–3] Increased IL-8 levels have been demonstrated in

CD, UC and pouchitis tissue, and these levels correlated with disease activity.[15,34] These studies were recently confirmed by Patel *et al.* and Sher *et al.*, who also reported elevated levels of IL-8 in non-inflamed tissue from ileoanal pouches and CD.[14,30] The role of IL-1 and TNFα in regulating IL-8 in colitis was demonstrated by Cominelli's group in the study referred to above.[16] They found that an inhibitor of the synthesis of IL-1 and TNFα (CGP 47969A) reduced the severity of damage in a rabbit model of colitis and this reduction of damage was associated with decreased colonic IL-1 and IL-8 levels.[16]

IL-8 production by isolated colonic crypt cells is increased in IBD.[35] The short chain fatty acid butyrate was shown to significantly reduce IL-8 secretion from these cells, possibly explaining the efficacy of short chain fatty acids in the treatment of distal UC and diversion colitis.[35]

### Tumour necrosis factor-α (TNFα)

Like IL-1, TNFα is released from macrophages early in the inflammatory response. TNFα has numerous roles in the inflammatory cascade and has been implicated in the pathogenesis of IBD (Table 1). Numerous studies have documented marked elevations TNFα in CD, UC and pouchitis.[3,8,14,30,36–38] Tissue, serum and intraluminal concentrations of TNFα have been shown to correlate with disease activity in both UC and CD.[3,36,37] Soluble TNFα receptors (sTNF-R), p55 and p75, have previously been shown to be elevated in the serum of patients with CD.[39] More recently, p55 and p75 were noted to be elevated in the urine of patients with either CD or UC, and these levels closely correlated with disease activity.[40] Thus, it is possible that intraluminal, serum or urine levels of TNFα or sTNF-R may aid in assessing and managing the IBD patient.

Recently, therapy aimed at regulation of TNFα activity has been evaluated. A single administration of an anti-TNFα monoclonal antibody (cA2) was found to be efficacious in 10 patients with CD who were resistant to conventional therapy.[41] In this open label, uncontrolled study, the mean disease severity (CD activity index) had decreased by 55% at 2 weeks and by 73% at 8 weeks following therapy. Endoscopy at 4 weeks' post-cA2 infusion showed near-complete healing of all ulceration. The average duration of response following a single dose of cA2 was 4 months. The extraintestinal manifestations of IBD (two with arthritis and one with pyoderma gangrenosum) also improved dramatically following cA2 administration. A marked reduction of IL-6 and C-reactive protein levels was observed, with the levels remaining below baseline values for up to 6 weeks following cA2 administration.[41] In a smaller study (five patients), clinical improvement of CD following treatment with cA2 appeared to correlate with a reduction in levels of cytokines associated with Th1 lymphocytes.[42]

A different form of anti-TNFα antibody (CDP571) was also recently evaluated. Two weeks following a single administration of CDP571, the mean CD activity index had decreased from 263 to 167, with six of the 15 patients achieving 'remission' (defined as a CD activity index of less than 150).[43]

A multicentre trial of cA2 is presently underway. In preliminary studies, a single dose of 1 to 20 mg/kg induced a significant clinical response in 18 of 20 patients. The dose given did not affect the likelihood of response, but the duration of the response appeared to be dose-dependent.[44] A role for cA2 in the treatment of UC remains equivocal at this stage. There has been one report of this antibody being effective in steroid-refractory UC,[45] while in another study cA2 was found to have no effect on reducing the severity of colonic inflammation in UC.[46]

The main criticisms of the studies performed to date using anti-TNFα antibodies are the small sample size and, from a mechanistic point of view, how a single infusion of cA2 can have such long-lasting effects. There are some indications that anti-TNFα antibodies may cause a reduction in specific lymphocyte subsets and thus may act by destroying these cells rather than blocking TNFα. Interestingly, similar results have been noted with the use of cA2 in the treatment of rheumatoid arthritis.[47]

### Interferons (IFN)

Interferons α, β and γ are part of a family of secreted proteins with potent antiproliferative and immunomodulatory activities.[1–3] Mononuclear cells isolated from the lamina propria of CD patients have been shown to have increased levels of both IFNγ and IFNα mRNA.[48] Since there have been reports of both improvement and exacerbation of IBD in patients undergoing IFNα therapy for other illnesses, two studies addressed the use of IFNα in CD. Davidsen *et al.* reported that only two of five CD patients treated with IFNα had significant reductions in the severity of their disease and all had influenza-like symptoms related to therapy.[49] Gasche *et al.* found that in a group of 12 patients with CD that received IFNα and prednisolone for 12 weeks followed by IFNα alone for another 12 weeks, none achieved remission and 66% of patients had be withdrawn from trial prematurely.[50] IFNα therapy was associated with significant side effects, failed to show any beneficial effect on serum IL-6 and acute phase protein levels and did not decrease the endoscopic severity of the disease.[50] Thus, based on the results from these two small studies, there does not appear to be any therapeutic role for IFNα in the treatment of CD.

### Anti-inflammatory Cytokines

The main anti-inflammatory cytokines include IL-1ra, IL-4, IL-10, IL-11, IL-13 and TGF-β (Table 2). IL-1ra has been discussed above.

### Interleukin-4 (IL-4)

IL-4 is primarily produced by mast cells and T lymphocytes. This cytokine has numerous actions as noted in Table 2, including the ability to down-regulate IFNγ, IL-1 and TNFα production and stimulate IL-1ra and IL-10 synthesis.[1,2,51] Recently it has been suggested that there is a defect in the ability of IL-4 to regulate the inflammatory response in IBD. Schreiber *et al.* noted that approximately 100-fold higher levels of IL-4 were required to inhibit monocyte production of IL-1β, TNFα and superoxide anion in patients with CD or UC compared with controls. IL-4 production by T-cells and the expression of IL-4 receptors by monocytes were reduced in patients with UC or CD. This could lead to impaired IL-4 responsiveness in these patients.[51,52]

### Interleukin-10 (IL-10)

In humans IL-10 is mainly produced by Th2 cells but it can also be produced by both Th1 cells and monocytes.[53–56] IL-10 has numerous actions but its main role appears to involve inhibition of macrophage and T cell function (Table 2).[53,56] There have been conflicting reports on IL-10 levels in IBD. In some studies IL-10 was found to be elevated in UC but not CD, suggesting UC had a Th2 pattern (increased IL-4, IL-5, IL-6 and IL-10) of cytokine activation whereas CD displayed a Th1 pattern (increased IL-2 and IFNγ).[1,2,53] Kucharzik *et al.* noted that IL-10 was elevated in the serum of patients with either UC or CD, and the levels of IL-10 correlated well with disease activity.[53] There was no difference in the serum levels of IL-10 from those with active UC compared with those with active CD.[53] The only difference noted was that IL-10 levels correlated well with serum IL-6 and soluble IL-2 receptor levels in those with

**Table 2.** Actions of anti-inflammatory cytokines and relevance to IBD

| Cytokine | Actions on cells and mediators | Relevance to IBD |
|---|---|---|
| IL-1ra | Endogenous receptor antagonist of IL-1 | Reduced severity of colitis in experimental model<br>Ratio of IL-1 to IL-1ra production is elevated in active UC and CD |
| IL-4 | Promotes<br>• mast cell growth<br>• IgE production<br>• IL-1ra and IL-10 synthesis<br>Inhibits<br>• TNFα and IFNγ synthesis | Decreased responsiveness of monocytes to IL-4 in CD and UC<br>Decreased IL-4 receptor expression in UC and CD |
| IL-10 | Inhibits<br>• TNFα, IL-1, IL-2, IL-6, IL-8, IFNγ and nitric oxide production<br>• macrophage and T lymphocyte function | IL-10 knockout mice develop enterocolitis<br>IL-10 reduced severity of experimental colitis |
| IL-11 | Promotes<br>• neutrophil, erythrocyte and platelet production<br>• proliferation and differentiation of small intestinal crypt cells | Reduces inflammation in several experimental models of colitis |
| IL-13 | Downregulates<br>• monocyte production of IL-1, IL-6, IL-8 and TNFα | Reduces severity of experimental colitis and eicosanoid production |
| TGFβ | Promotes<br>• wound healing, epithelial restitution<br>• IL-1ra synthesis<br>Inhibits<br>• nitric oxide synthesis<br>• TNFα, IFNγ synthesis<br>• leukocyte rolling (E selectin expression) | Increased TGF-β levels in IBD<br>TGF-β knockout mice develop colitis |

Abbreviations: UC, ulcerative colitis; CD, Crohn's disease; IBD, inflammatory bowel disease.

CD but not in UC patients.[53] The theory that IL-10 regulation is significantly different in CD versus UC was further disputed by the findings of Schreiber *et al.*[55] They found no difference in the tissue concentrations of IL-10 in patients with CD versus those with UC.[55] It was also noted that the IL-10 downregulation of TNF$\alpha$ and IL-1$\beta$ secretion by peripheral monocytes was similar in patients with IBD and normal controls.[55] As noted above, patients with IBD have an abnormal IL-1:IL-1ra ratio in favour of increased IL-1 activity. In the study by Schreiber's group, the addition of IL-10 to either peripheral monocytes or intestinal macrophages from patients with IBD could restore the IL-1:IL-1ra ratio to that found in cell suspensions from normal controls.[55] Thus, it was hypothesized that IL-10 regulation was intact in IBD and that there was not a true deficiency of IL-10, but there may be a deficiency of IL-10 relative to the degree of inflammation in IBD. Schreiber *et al.* treated three patients with active UC with IL-10 enemas and all exhibited marked reductions in TNF$\alpha$ and IL-1$\beta$ secretion, but only a minimal reduction in stool frequency and endoscopic scores.[55] A multicentre trial of the use of IL-10 in CD is presently underway.

There is emerging evidence from animal models for a key role of IL-10 in the maintenance of intestinal mucosal integrity and mucosal immune function. Mice in which the gene for IL-10 has been deleted exhibit profound enterocolitis, which can be prevented through administration of recombinant IL-10.[54] IL-10 can also reduce the severity of colitis in a number of experimental models, although there appears to be some variation in the responsiveness of each model and of the dose required to achieve a significant effect.[57–59] IL-10 has also recently been shown to prevent secretagogue-induced epithelial secretion in the rat small intestine, suggesting that it might be an important factor in limiting the development of diarrhoea.[60]

### Interleukin-11 (IL-11)

IL-11 was originally isolated from bone marrow stromal cell lines and was found to have both haematopoietic and non-haematopoietic regulatory actions.[61,62] IL-11 receptor belongs to the same family of cytokine receptors as that for IL-6. Both IL-6 and IL-11 stimulate T cell-dependent B cell maturation and enhance platelet and myeloid cell differentiation.[61,62] The mechanisms involved in the anti-inflammatory actions of IL-11 are not well understood. It has been found to decrease to the severity of intestinal damage following irradiation, cytoablative drugs or exposure to *Clostridium difficile* toxin A.[63–65] IL-11 has also been found to decrease the severity of inflammation in the TNBS, acetic acid and transgenic HLA-B27 models of colitis.[66–68] The role of IL-11 in the inflammatory response in UC and CD has yet to be determined.

### Interleukin-13 (IL-13)

IL-13 is a lymphocyte-derived cytokine that appears to have some immunosuppressive actions that are similar to IL-4 and IL-10. IL-13 downregulates the release of IL-1$\beta$, IL-6, IL-8 and TNF$\alpha$ from monocytes, and also induces the expression of vascular cell adhesion molecule (VCAM-1). On the other hand, IL-13 can inhibit IFN-induced upregulation of intercellular adhesion molecule-1 (ICAM-1).[69–73] Nitric oxide (NO) synthesis and inducible nitric oxide synthase (iNOS) are increased in the colonic epithelium of patients with UC.[74] In a recent study using a human colonic epithelial cell line, IL-13 downregulated NO release and iNOS expression but IL-10 had no effect.[75] Both IL-4 and IL-13 have been reported to suppress cyclooxygenase-2 expression in osteoblasts, but this interaction has not been studied in the gut.[70] In peripheral monocytes isolated from IBD patients the ability of IL-13 to inhibit the production of pro-inflammatory cytokines was reduced compared with cells from control patients or from patients with inactive IBD.[76] Interestingly, it appears that IL-10 can act synergistically with IL-4 or IL-13 to downregulate pro-inflammatory cytokine production by peripheral blood monocytes in IBD, suggesting that combined immunosuppressive cytokine therapy may be beneficial.[77] IL-13 is also produced by activated mast cells, but the implications of this to IBD has yet to be studied.[78]

### Transforming growth factor-$\beta$ (TGF-$\beta$)

TGF-$\beta$ is a cytokine with several anti-inflammatory properties, including modulating the function and secretory activity of macrophages and other immunocytes.[1] TGF-$\beta$ is also a promoter of wound healing.[1] There appears to be a significant upregulation of TGF-$\beta$ release in patients with IBD and following intestinal irradiation (Table 2).[1,79–82] TGF-$\beta$ can downregulate intestinal cell proliferation and promote cellular differentiation.[80] TGF-$\beta$ can also modulate the production of other cytokines, particularly IL-1ra (increased), IL-1, TNF$\alpha$ and IFN-$\gamma$ (decreased),[83] can suppress nitric oxide production by macrophages[84] and can reduce leukocyte rolling on the vascular endothelium by inhibiting expression of E-selectin.[85]

Mice in which the gene for TGF-β has been deleted develop colitis, suggesting a key role for this cytokine in the maintenance of mucosal integrity and mucosal immune function.[86] More direct evaluation of the role of TGF-β in human IBD has not yet been performed.

### Conclusions

While incompletely understood, the pathogenesis of IBD is generally believed to be related to an improperly regulated mucosal immune response to luminal microbes. As central regulators of immune responses, the cytokine family have been extensively studied in human IBD and experimental models of colitis. The observation that severe inflammation of the small and/or large intestine occurs spontaneously in several mouse models in which the genes for various cytokines have been deleted adds support to the hypothesis that perturbations in the cytokine cascade could contribute to the development of IBD. Clinical studies of IBD have begun to reveal marked alterations in the production of certain cytokines, or in the responsiveness of immunocytes to various cytokines. Ultimately, these studies may help to identify potential targets for the therapy of these diseases. Indeed, the early, very promising results with antibodies directed against TNFα suggest that cytokine-targeted therapy may in the future become a mainstream treatment for IBD, or at the very least, may be useful adjuncts to existing therapies in the management of IBD in certain subsets of patients.

### References

1. Cominelli F. Cytokines. In: Wallace JL, ed. *Immunopharmacology of the Gastrointestinal System*. London: Academic Press, 1993; 123–136.
2. Kam L, Pizarro TT, Cominelli F. Cytokine and chemokines in inflammatory bowel disease. *Curr Opin Gastroenterol* 1995; **11**: 305–309.
3. Sartor RB. Cytokines in intestinal inflammation: pathophysiologic and clinical considerations. *Gastroenterology* 1994; **106**: 533–539.
4. Ligumsky M, Simon PL, Karmeli F, Rachmilewitz D. Role of interleukin 1 in inflammatory bowel disease: enhanced production during active disease. *Gut* 1990; **31**: 686–689.
5. Nishiyama T, Mitsuyama K, Toyonaga A, Sasaki E, Tanikawa K. Colonic mucosal interleukin 1 receptor antagonist in inflammatory bowel disease. *Digestion* 1994; **55**: 368–373.
6. Mazlam MZ, Hodgson HJ. Interrelations between interleukin-6, interleukin-1 beta, plasma C-reactive protein values, and *in vitro* C-reactive protein generation in patients with inflammatory bowel disease. *Gut* 1994; **35**: 77–83.
7. Reinecker HC, Steffen M, Witthoeft T, Pflueger I, Schreiber S, MacDermott RP, Raedler A. Enhanced secretion of tumor necrosis factor-alpha, IL-6, and IL-1 beta by isolated lamina propria mononuclear cells from patients with ulcerative colitis and Crohn's disease. *Clin Exp Immunol* 1993; **94**: 174–181.
8. Casini-Raggi V, Kam L, Chong YJ, Fiocchi C, Pizarro TT, Cominelli F. Mucosal imbalance of IL-1 and IL-1 receptor antagonist in inflammatory bowel disease. A novel mechanism of chronic intestinal inflammation. *J Immunol* 1995; **154**: 2434–2440.
9. Hyams JS, Fitzgerald JE, Wyzga N, Treen WR, Justinich CJ, Kreutzer DL. Characterization of circulating interleukin-1 receptor antagonist expression in children with inflammatory bowel disease. *Dig Dis Sci* 1994; **39**: 1893–1899.
10. McCall RD, Haskill S, Zimmermann EM, Lund PK, Thompson RC, Sartor RB. Tissue interleukin 1 and interleukin 1 receptor antagonist expression in enterocolitis in resistant and susceptible rats. *Gastroenterology* 1994; **106**: 960–972.
11. Cominelli F, Nast CC, Clark BD, Schindler R, Llerena R, Eysselin VE, Thompson RC, Dinarello CA. Interleukin 1 (IL-1) gene expression, synthesis, and effect on specific IL-1 receptor blockade in rabbit immune complex colitis. *J Clin Invest* 1990; **86**: 972–980.
12. McCafferty D-M, Rioux KP, Wallace JL. Granulocyte infiltration in experimental colitis is interleukin-1 dependent and leukotriene independent. *Eicosanoids* 1992; **5**: 121–125.
13. Ferretti M, Casini-Raggi V, Pizarro TT, Eisenberg S, Nast CC, Cominelli F. Neutralization of endogenous IL-1 receptor antagonist exacerbates and prolongs inflammation in rabbit immune colitis. *J Clin Invest* 1994; **94**: 449–453.
14. Sher ME, D'Angelo AJ, Stein TA, Bailey B, Burns G. Cytokines in Crohn's colitis. *Am J Surg* 1995; **169**: 133–136.
15. Gionchetti P, Campieri M, Belluzzi A, Bertinelli E, Ferretti M, Brignola C, Poggioli G, Miglioli M, Barbara L. Mucosal concentrations of interleukin-1 beta, interleukin-6, interleukin-8, and tumor necrosis factor-alpha in pelvic ileal pouches. *Dig Dis Sci* 1994; **39**: 1525–1531.
16. Casini-Raggi V, Monsacchi L, Vosbeck K, Nast CC, Pizarro TT, Cominelli F. Anti-inflammatory effects of CGP 47969A, a novel inhibitor of proinflammatory cytokine synthesis, in rabbit immune colitis. *Gastroenterology* 1995; **109**: 812–818.
17. Pullan RD, Rhodes J, Ganesh S, Mani V, Morris JS, Williams GT, Newcombe RG, Russel MA, Feyerabend C, Thomas GA. Transdermal nicotine for active ulcerative colitis. *New Engl J Med* 1994; **330**: 811–815.
18. Thomas GA, Rhodes J, Mani V, Williams GT, Newcombe RG, Russel MA, Feyerabend C. Transdermal nicotine as maintenance therapy for ulcerative colitis. *New Engl J Med* 1995; **332**: 988–992.
19. Van Dijk JPM, Madretsma GS, Keuskamp ZJ, Zijlstra FJ. Nicotine inhibits cytokine synthesis by mouse colonic mucosa. *Eur J Pharmacol* 1995; **278**: R11–R12.
20. Manzano L, Alvarez-Mon M, Vargas JA, Giron JA, Fernandez-Corugedo A, Roman LI, Albarran F, Durantez A. Deficient interleukin 2 dependent proliferation pathway in T lymphocytes from active and inactive ulcerative colitis patients. *Gut* 1994; **35**: 955–960.
21. Mullin GE, Lazenby AJ, Harris ML, Bayless TM, James SP. Increased interleukin-2 messenger RNA in the intestinal mucosal lesions of Crohn's disease but not ulcerative colitis. *Gastroenterology* 1992; **102**: 1620–1627.
22. Gurbindo C, Sabbah S, Menezes J, Justinich C, Marchand R, Seidman EG. Interleukin-2 production in pediatric inflammatory bowel disease: evidence for dissimilar mononuclear function in Crohn's disease and ulcerative colitis. *J Ped Gastroenterol Nutr* 1993; **17**: 247–254.
23. Sparano JA, Brandt LJ, Dutcher JP, DuBois JS, Atkins MB. Symptomatic exacerbation of Crohn's disease after treatment with high dose interleukin-2. *Ann Int Med* 1993; **118**: 617–618.
24. Brynskov J, Freund J, Norby Rasmussen S, *et al.* Final report on a placebo controlled, double-blind, randomized, multicentre trial of cyclosporin treatment in active Crohn's disease. *Scand Gastroenterol* 1991; **26**: 689–695.
25. Lichtiger S, Present DH, Kornbluth A, Gelernt I, Bauer J, Galler G, Michelassi F, Hanauer S. Cyclosporine in severe ulcerative colitis refractory to steroid therapy. *New Engl J Med* 1994; **330**: 1841–1845.
26. Aiko S, Grisham MB. Effects of cyclosporine of FK506 on pathophysiology observed in a model of chronic granulomatous colitis in rats. *Gastroenterology* 1995; **108**: A768 (abstract).
27. Dubucquoi S, Janin A, Klein O, Desreumaux P, Quandalle P, Cortot A, Capron M, Colombel J-F. Activated eosinophils and interleukin 5 expression in early recurrence of Crohn's disease. *Gut* 1995; **37**: 242–246.
28. Stevceva L, Pavli P, Matthei K, Yound I, Doe WF. Dextran sulphate sodium-induced colitis develops in IL-5 knockout mice. *Gastroenterology* 1995; **108**: A922 (abstract).
29. Baumann H, Gauldie J. The acute phase response. *Immunol Today* 1994; **15**: 74–80.
30. Patel RT, Bain I, Youngs D, Keighly MR. Cytokine production in pouchitis is similar to that in ulcerative colitis. *Dis Colon Rectum* 1995; **38**: 831–837.
31. Holtkamp W, Stollberg T, Reis HE. Serum interleukin-6 is related to disease activity but not specificity in inflammatory bowel disease. *J Clin Gastroenterol* 1995; **20**: 123–126.
32. Mitsuyama K, Toyonaga A, Sasaki E, Ishida O, Ikeda H, Tsuruta O, Harada K, Tateishi H, Nishiyama T, Tanikawa K. Soluble interleukin-6 receptors in inflammatory bowel disease: relation to circulating interleukin-6. *Gut* 1995; **36**: 45–49.
33. Louis E, Belaiche J, Van Kemseke C, DeGroote D. Crohn's disease relapse prediction by interleukin-6 serum level. *Gastroenterology* 1995; **108**: A865 (abstract).
34. Mitsuyama K, Toyonaga A, Sasaki E, Watanabe K, Tateishi H, Nishirama

T, Saiki T, Ikeda H, Tsudruta O, Tanikawa K. IL-8 as an important chemoattractant for neutrophils in ulcerative colitis and Crohn's disease. *Clin Exp Immunol* 1994; **96**: 432–436.
35. Gibson P, Rosella O. Interleukin-8 secretion by colonic crypt cells *in vitro*: response to injury suppressed by butyrate and enhanced in inflammatory bowel disease. *Gut* 1995; **37**: 536–543.
36. Casellas F, Papo M, Guarner F, Antolin M, Armengol JR, Malagelada JR. Intraluminal colonic release of immunoreactive tumour necrosis factor in chronic ulcerative colitis. *Clin Sci* 1994; **87**: 453–458.
37. Raab Y, Sunberg C, Hallgren R, Knutson L, Gerdin B. Mucosal synthesis and release of prostaglandin $E_2$ from activated eosinophils and macrophages in ulcerative colitis. *Am J Gastroenterol* 1995; **90**: 614–620.
38. Autschbach F, Schurmann G, Quio L, Merz H, Wallich R, Meuer SC. Cytokine messenger RNA expression and proliferation status of intestinal mononuclear cells in noninflamed gut and Crohn's disease. *Virchow's Archiv* 1995; **426**: 51–60.
39. Hudson M, Gallati H, Ryff JC, Pounder RE, Wakefield AJ. Serum tumour necrosis factor (TNF) and soluble TNF receptors p55 and p75 in Crohn's disease. *Gastroenterology* 1993; **104**: A715 (abstract).
40. Hadziselimovic F, Emmons LR, Gallati H. Soluble tumour necrosis factor receptors p55 and p75 in the urine monitor disease activity and the efficacy of treatment of inflammatory bowel disease. *Gut* 1995; **37**: 260–263.
41. Van Dullemen HM, Van Deveter SJH, Hommes DW, Bijl HA, Jansen J, Tytgat GNJ, Wood J. Treatment of Crohn's disease with anti-tumour necrosis factor chimeric antibody (cA2). *Gastroenterology* 1995; **109**: 129–135.
42. Plevy SE, Carramanzana NM, Deem RL, Woody JN, Targan SR. Clinical improvement in Crohn's disease patients treated with anti-TNFα correlates with downregulated mucosal T helper 1 responses. *Gastroenterology* 1996; **110**: A993 (abstract).
43. Stack W, Mann S, Roy A, Heath P, Sopwith M, Freeman J, Holmes G, Long R, Forbes A, Kamm M, Hawkey C. The effects of CDP571, an engineered human $IgG_4$ anti-TNFα antibody in Crohn's disease. *Gastroenterology* 1996; **110**: A1018 (abstract).
44. McCabe RP, Woody J, van Deveter S, Targan SR, Mayer L, van Hogezand R, Rutgeerts P, Hanauer SB, Podolsky D, Elson CO. A multicenter trial of cA2 anti-TNF chimeric monoclonal antibody in patients with active Crohn's disease. *Gastroenterology* 1996; **110**: A962 (abstract).
45. Sands BE, Podolsky DK, Tremaine WJ, Sanborn WJ, Rutgeerts PJ, Hanauer SB, Mayer L, Targan SR, DeWoody KL, Braakman TAJ, Woody JN. Chimeric monoclonal anti-tumor necrosis factor antibody cA2 in the treatment of severe, steroid-refractory ulcerative colitis. *Gastroenterology* 1996; **110**: A1008 (abstract).
46. Baert F, D'Haens G, Geboes K, Ectors N, Rutgeerts P. TNF-α antibody therapy causes a fast and dramatic decrease of histological colonic inflammation in Crohn's disease but not in ulcerative colitis. *Gastroenterology* 1996; **110**: A859 (abstract).
47. Elliot MJ, Maini RN, Feldmann M, Kalden JR, Antoni C, Smolen JS, Leeb B, Breedveld FC, Macfarlane JD, Bijl H, Woody JN. Randomized double-blind comparison of chimeric monoclonal antibody to tumour necrosis factor alpha (cA2) versus placebo in rheumatoid arthritis. *Lancet* 1994; **344**: 1105–1110.
48. Fais S, Capobiachi MR, Silvestri M, Mercuri F, Pallone F, Dianzani F. Interferon expression in Crohn's disease patients: increased interferon-gamma and alpha mRNA in the intestinal lamina propria mononuclear cells. *J Interferon Res* 1994; **14**: 235–238.
49. Davidsen B, Munkholm P, Schlichting P, Nielsen OH, Krarup H, Bonnevie-Nielsen V. Tolerability of interferon alpha-2b, a possible new treatment of active Crohn's disease. *Aliment Pharm Therap* 1995; **9**: 75–79.
50. Gasche C, Reinisch W, Vogelsang H, Potzi R, Markis E, Micksche M, Wirth HP, Gangl A, Lochs H. Prospective evaluation of interferon-alpha in treatment of chronic active Crohn's disease. *Dig Dis Sci* 1995; **40**: 800–804.
51. Schreiber S, Heinig T, Panzer U, Reinking R, Bouchard A, Stahl PD, Raedler A. Impaired response of activated mononuclear phagocytes to interleukin 4 in inflammatory bowel disease. *Gastroenterology* 1995; **108**: 21–33.
52. Karttrunnen R, Breese EJ, Walker-Smith JA, MacDonald TT. Decreased mucosal interleukin-4 (IL-4) production in gut inflammation. *J Clin Pathol* 1994; **47**: 1015–1018.
53. Kucharzik T, Stoll R, Lugering N, Domschke W. Circulating antiinflammatory IL-10 in patients with inflammatory bowel disease (IBD). *Clin Exp Immunol* 1995; **100**: 452–456.
54. Kühn R, Löhler J, Rennick D, Rajewsky K, Müller W. Interleukin-10 deficient mice develop chronic enterocolitis. *Cell* 1993; **75**: 263–274.
55. Schreiber S, Heinig T, Thiele HG, Raedler A. Immunoregulatory role of interleukin 10 in patients with inflammatory bowel disease. *Gastroenterology* 1995; **108**: 1434–1444.
56. Del Prete G, De Carli M, Amerigogna F, Giudizi MG, Biagotti R, Romagnini S. Human IL-10 is produced by both type 1 (Th 1) and type 2 helper (Th 2) T cell clones and inhibits their antigen-specific proliferation and cytokine production. *J Immunol* 1993; **150**: 353–360.
57. Ribbons KA, Eloby-Childress S, Thompson J, Zhang XJ, Pennline K, Miller MJS. Effect of interleukin-10 in TNBS-induced colitis in rats. *Gastroenterology* 1996; **110**: A11001.
58. Grool TA, Meenan J, Van Dulleman H, Koster F, Ten Kate FJW, Lebeaut A, Tytgat GNJ, Van Deventer SJH. The anti-inflammatory effect of interleukin 10 in a rabbit model of immune complex-induced colitis. *Gastroenterology* 1996; **110**: A918.
59. Herfarth H, Janardhanam R, Rath HC, Sartor RB. In vivo IL-10 treatment suppresses experimental chronic granulomatous inflammation and has an additive effect with corticosteroids. *Gastroenterology* 1996; **110**: A924.
60. Madsen KL, Tavernini MM, Fedorak RN. Interleukin-10 modulates ion transport in rat small intestine. *Gastroenterology* 1996; **111**: 936–944.
61. Kobayashi S, Teramura M, Oshimi K, Mizoguchi H. Interleukin-11. *Leukemia Lymphoma* 1994; **17**: 45–49.
62. Neben S, Turner K. The biology of interleukin-11. *Stem Cells* 1993; **11** (suppl 2): 156–162.
63. Potten CS. Interleukin-1 protects to clonogenic stem cells in murine small-intestinal crypt from impairment of their reproductive capacity by radiation. *Int J Cancer* 1995; **62**: 356–361.
64. Du XX, Doerschuk CM, Orazi A, Williams DA. A bone marrow stromal-derived growth factor, interleukin-11, stimulates recovery of small intestinal mucosal cells after cytoablative therapy. *Blood* 1994; **83**: 33–37.
65. Castagliuolo I, LaMont JT, Baker C, Nikulasson ST, Keith JC, Pothoulakis C. Recombinant human interleukin-11 (rhIL-11) inhibits *Clostridium difficile* toxin A-mediated enterotoxicity in rat ileum. *Gastroenterology* 1995; **108**: A792 (abstract).
66. Albert LM, Ferranti TJ, Erickson JE, Donnelly LH, Schaub RG, Keith JC. Dose response and schedule studies of recombinant human interleukin-11 in acetic acid-induced colonic injury in rats. *Gastroenterology* 1995; **108**: A768 (abstract).
67. Keith JC, Albert LM, Ferranti TJ, Erickson JE, Mason LE, Donnelly LH, Misra BR, Schaub RG. Recombinant human interleukin-11 (rhIL-11) decreases inflammatory bowel disease in HLA-B27 transgenic rats. *Gastroenterology* 1995; **108**: A846 (abstract).
68. Qiu B, Pfeiffer CJ. Protection by recombinant human interleukin-11 against experimental TNBS-induced colitis in rats. *Dig Dis Sci* 1996; **41**: 1625–1630.
69. Yano S, Sone S, Nishioka Y, Mukaida N, Matsushima K, Ogura T. Differential effects of anti-inflammatory cytokine (IL-4, IL-10, IL-13) on tumoricidal and chemotactic properties of human monocytes induced by monocyte chemotactic and activating factor. *J Leuk Biol* 1995; **57**: 303–309.
70. Onoe Y, Miyaura C, Kaminakayashiki T, Nagai Y, Noguchi K, Chen QR, Seo H, Ohta H, Nozawa S, Kudo I, Suda T. IL-13 and IL-4 inhibit bone reabsorption by suppressing cyclooxygenase-2-dependent prostaglandin synthesis in osteoblasts. *J Immunol* 1996; **156**: 758–764.
71. Kim C, Schinkel C, Fuchs D, Satdler J, Walz A, Zedler S, von Donnersmarck GH, Faist E. Interleukin-13 effectively down-regulates the monocyte inflammatory potential during traumatic stress. *Arch Surg* 1995; **130**: 1330–1336.
72. Bochner BS, Klunk DA, Sterbinsky SA, Coffman RL, Schleimer RP. IL-13 selectively induces vascular cell adhesion molecule-1 expression in human endothelial cells. *J Immunol* 1995; **154**: 799–803.
73. Schlaak JF, Schwarting A, Knolle P, Meyer zum Buschenfelde KH, Mayet W. Effects of Th1 and Th2 cytokines on cytokine production and ICAM-1 expression on synovial fibroblasts. *Ann Rheumatic Dis* 1995; **54**: 560–565.
74. Boughton-Smith NK, Evans SM, Hawkey CJ, Cole AT, Balsitis M, Whittle BJR, Moncada S. Nitric oxide synthase activity in ulcerative colitis and Crohn's disease. *Lancet* 1993; **342**: 338–340.
75. Kolios G, Robertson DAF, Westwick J. Interleukin-13 regulates the nitric oxide production in human colonic epithelial cells. Comparison with interleukin-4 and interleukin-10. *Gastroenterology* 1996; **110**: A940 (abstract).
76. Kucharzik T, Lugering N, Weigelt H, Adolf M, Domschke W, Stoll R. Comparison of the suppressive effects of interleukin-13, interleukin-10, and interleukin-4 on peripheral blood monocytes from patients with IBD. *Gastroenterology* 1996; **110**: A943 (abstract).
77. Kucharzik T, Lugering N, Stoll R. Synergistic effect of immunoregulatory cytokines on peripheral blood monocytes: potential therapeutic approach in IBD? *Gastroenterology* 1996; **110**: A943 (abstract).
78. Burd PR, Thompson WC, Max EE, Mills FC. Activated mast cells produce interleukin 13. *J Exp Med* 1995; **181**: 1373–1380.
79. Potten CS, Owen G, Hewitt D, Chadwick CA, Hendry H, Lord BI, Woolford LB. Stimulation and inhibition of proliferation in the small intestinal crypts of the mouse after *in vivo* administration of growth factors. *Gut* 1995; **36**; 864–873.
80. Booth C, Evans GS, Potten CS. Growth factor regulation of proliferation in primary cultures of small intestinal epithelium. *In Vitro Cell Develop Biol* 1995; **31**: 234–243.
81. McCabe RP, Secrist H, Botney M, Egan M, Peters MG. Cytokine mRNA expression in intestine from normal and inflammatory bowel disease patients. *Clin Immunol Immunopath* 1993; **66**: 52–58.
82. Babyatsky MW, Rossiter G, Podolsky DK. Expression of transforming growth factors α and β in colonic mucosa in inflammatory bowel disease. *Gastroenterology* 1996; **110**: 975–984.
83. Turner M, Chantry C, Katsidis P. Induction of the interleukin-1 receptor antagonist protein by transforming growth factor-β. *Eur J Immunol* 1991; **21**: 1635–1639.
84. Vodovotz Y, Bogdan C, Paik J, Xie Q, Nathan C. Mechanisms of suppression of macrophage nitric oxide release by transforming growth factor β. *J Exp Med* 1993; **178**: 605–613.
85. Gamble JR, Khew-Goodall Y, Vadas MA. Transforming growth factor-β inhibits E-selectin expression on human endothelial cells. *J Immunol* 1993; **150**: 4494–4503.
86. Kulkarni AB, Karlsson S. Transforming growth factor-$\beta_1$ knockout mice: a mutation in one cytokine gene causes a dramatic inflammatory disease. *Am J Pathol* 1993; **143**: 3–9.

ACKNOWLEDGEMENTS. P.L.B. is supported by a Medical Research Council of Canada (MRC) Clinician Scientist award. J.L.W. is a MRC Senior Scientist and an Alberta Heritage Foundation for Medical Research Scientist.

**Received 21 October 1996; accepted 17 December 1996**
